# Aberrant topology of white matter networks in patients with methamphetamine dependence and its application in support vector machine-based classification

**DOI:** 10.1038/s41598-023-33199-8

**Published:** 2023-04-28

**Authors:** Ping Cheng, Yadi Li, Gaoyan Wang, Haibo Dong, Huifen Liu, Wenwen Shen, Wenhua Zhou

**Affiliations:** 1 Department of Radiology, Ningbo Medical Treatment Center Lihuili Hospital, Ningbo University, 57# Xing Ning Road, Ningbo, Zhejiang China; 2 Department of Psychiatry, Ningbo Kangning Hospital, Ningbo University, 1# Zhuangyu South Road, Ningbo, Zhejiang China

**Keywords:** Diseases of the nervous system, Neurology

## Abstract

Brain white matter (WM) networks have been widely studied in neuropsychiatric disorders. However, few studies have evaluated alterations in WM network topological organization in patients with methamphetamine (MA) dependence. Therefore, using machine learning classification methods to analyze WM network topological attributes may give new insights into patients with MA dependence. In the study, diffusion tensor imaging-based probabilistic tractography was used to map the weighted WM networks in 46 MA-dependent patients and 46 control subjects. Using graph-theoretical analyses, the global and regional topological attributes of WM networks for both groups were calculated and compared to determine inter-group differences using a permutation-based general linear model. In addition, the study used a support vector machine (SVM) learning approach to construct a classifier for discriminating subjects with MA dependence from control subjects. Relative to the control group, the MA-dependent group exhibited abnormal topological organization, as evidenced by decreased small-worldness and modularity, and increased nodal efficiency in the right medial superior temporal gyrus, right pallidum, and right ventromedial putamen; the MA-dependent group had the higher hubness scores in 25 regions, which were mainly located in the default mode network. An SVM trained with topological attributes achieved classification accuracy, sensitivity, specificity, and kappa values of 98.09% ± 2.59%, 98.24% ± 4.00%, 97.94% ± 4.26%, and 96.18% ± 5.19% for patients with MA dependence. Our results may suggest altered global WM structural networks in MA-dependent patients. Furthermore, the abnormal WM network topological attributes may provide promising features for the construction of high-efficacy classification models.

## Introduction

Substance use disorder refers to a range of abnormal behaviors associated with use of psychoactive substances that alter normal brain activity and have a wide range of effects on an individual's health^1^. Use of methamphetamine (MA), a synthetic drug widely abused globally, is growing rapidly. According to the 2020 World Drug Report by the United Nations, which included data up to 2018, the total number of MA abusers was approximately 27 million, which ranked second to marijuana in illegal drug use worldwide. The Annual Report on the National Narcotic Control Commission (NNCC) of China showed that 1.35 million of 2.4 million current drug users were MA abusers, which accounted for 56.1% through 2021. Moreover, excessive use of MA causes serious health consequences and is associated with high crime rates^2^.

Diffusion tensor imaging (DTI), a new technology developed and optimized based on diffusion-weighted imaging, has allowed for the noninvasive study of the orientation and integrity of white matter (WM) fiber bundles^3^. Information about WM organization of brain microstructure can be obtained from fractional anisotropy (FA), mean diffusivity (MD), axial diffusivity (AD), etc. Among them, FA is considered to be an indicator of WM integrity, which may be related to the integrity of the axon membrane, the degree of myelination, neuronal fiber density, and fiber orientation. Diffusion tensor imaging has been widely used to study drug addiction and mental disorders^4,5^. In MA dependence-related research, have characterized changes in the brain microstructure of individuals addicted to MA at the molecular level based using DTI^6^, and reported abnormal WM integrity. However, the results of these studies have been inconsistent. Reduced FA in the frontal lobes of MA-dependent subjects has been reported in several studies^7,8^. Zhuang et al.^9^ have shown microstructural defects in WM surrounding the basal ganglia in MA-dependent subjects. Huang et al.^10^ found the extensive reduction of FA in WM in MA-dependent subjects using TBSS (tract-based spatial statistics). Significantly reduced FA values in these brain regions may be a result of demyelination or axonal damage^11^. Although these previous imaging studies provided valuable information on the anatomical characteristics of nerve fiber bundles in individuals with MAdependence, system-level understanding is limited. Therefore, we explored the effects of MA addiction on the brain at the system or network level.

The brain is an example of a complex network consisting of a large number of interacting components. Therefore, understanding the operation of the brain in realtime is a major challenge. Fortunately, complex networks are ubiquitous and can withstand detailed analysis. Common examples include transportation systems, social networks in the online and real-world, and the World Wide Web^12–14^. In the past two decades, the development of graph theory has begun to provide a conceptual framework for the study of the structural characteristics of complex networks. A key finding in this work is that the structural and functional networks of the brain share common features^15^, such as small-worldness, network hubs, and hierarchical modularity, with many other complex systems. The value of brain network research is reflected in a range of network changes found in neurological and psychiatric disorders, including epilepsy^16^, depression^17^, Alzheimer's disease^18^, schizophrenia^19^, and others. Moreover, there is evidence that specific pathological conditions are associated with changes in brain network topography^20–23^. Functional MRI studies of Alzheimer’s disease by Buckner et al.^24^ showed that amyloid deposition occurs preferentially in the locations of cortical hubs, and the level of functional connectivity across the brain was positively correlated with the level of amyloid deposition. However, few studies have reported the aberrant topological structure of brain functional networks in MA-dependent individuals^25,26^. Siyah et al.^26^ showed that the whole-brain resting-state functional networks in MA-dependent individuals were likely shifted toward the random organization. Analysis of EEG (electroencephalogram) data from 36 MA abusers by Khajehpour et al.^25^ suggested decreased characteristic path length and increased clustering coefficient in resting-state brain functional networks. However, MA dependence-related changes in brain structural connectivity and topological organization of brain WM networks require further characterization.

Although traditional univariate methods (such as t-tests and analysis of variance) used in previous studies can help to locate brain regions that differ significantly between groups, these traditional univariate methods have many limitations^27–29^: ① group-level analysis tends to ignore individual differences, and the results of the group-level analysis can only provide qualitative descriptions without prediction of individual performance; ② univariate analysis methods are susceptible to noise; ③ univariate methods cannot be used to perform joint analysis of the influence of multiple features, and further multiple comparisons are prone to generation of false positives. Multivariate analysis is an extension of univariate analysis and can be used to overcome some limitations of univariate analysis. Machine learning, which mainly uses multivariate analysis methods, looks for laws from a large amount of observed data and uses these laws to predict future data or unobservable data. There are many kinds of machine learning algorithms, including naive Bayes, k-nearest neighbors, decision trees, regression, support vector machines (SVM), and artificial neural networks. Among these, the most used in the field of neuroimaging is SVM, which seeks the best compromise between model complexity and learning ability according to the limited sample information to obtain the best generalization ability. Moreover, SVM excels at solving small sample, nonlinear, and high-dimensional pattern recognition problems, and can be extended to other machine learning problems such as function fitting. Support vector machine learning has been extensively used to analyze structural and functional MRI data for the classification of various substance use disorders^30^, such as MAdependence^31^, heroindependence^32^, cocainedependence^33^, and nicotinedependence^34^.

Abnormal brain network topological attributes, which identified novel potential biomarkers for the diagnosis of neurological diseases, have been found in various brain diseases, such as schizophrenia^35^ and Alzheimer’s disease^36^. However, few studies have focused on the use of topological attributes to diagnose MA dependencies. Based on these studies, we used DTI-based probabilistic tractography and graph theory to describe the WM networks of MA-dependent individuals, and an SVM was used to construct a classifier with WM network topological attributes that displayed significant inter-group differences, which was expected to discriminate individuals with MA dependence from controls.

We hypothesized that (1) MA-dependent individuals were likely to have altered global and regional topological attributes of whole-brain WM networks compared to controls; (2) since MA dependence can cause psychiatric symptoms including anxiety, depression, and hostile suspicion, we hypothesized that abnormal topological attributes of the brain in MA-dependent patients were significantly correlated with the severity of psychiatric symptoms; (3) WM network topological attributes could be used as effective features in constructing a classifier to identify MA-dependent individuals.

## Materials and methods

### Subjects

Forty-six right-handed, male, MA-dependent patients were recruited from Ningbo Kangning hospital, Ningbo, PR China. There were very few female MA-dependent patients at this hospital. Forty-six age and education-matched, right-handed, healthy male subjects were recruited as controls from local communities. The inclusion criteria for MA users were (1) meeting the Diagnostic and Statistical Manual of Mental Disorders, Fourth edition, Text revision (DSM-IV-TR) criteria for current MA dependence. All patients received an MRI scan within 4 to 7 days after the time of last use of MA; and (2) no current, or history of, dependence on other drugs of abuse (except nicotine). The exclusion criteria included (1) a history of psychiatric illness, neurological disorder, or major chronic medical illnesses before MA use; and (2) having metallic or electronic devices or implants. The same inclusion and exclusion criteria were used for the normal controls, except these individuals had no history of drug abuse or dependence, other than nicotine.

The psychiatric symptoms of MA dependence were evaluated by 2 psychiatrists using the Brief Psychiatric Rating Scale (BPRS) and the Hamilton Anxiety Scale (HAMA) within the first 2 days of hospital admission, and 3 days prior to MRI scanning. The BPRS contains five factors: anxiety-depression, lack of vitality, activity, hostility-suspicion, and thinking disorder. The former factors reflect negative symptoms, the third and fourth reflect positive symptoms, and thinking disorders may be affected by both positive and negative symptoms.

This study was approved by the Institutional Review Board of Ningbo Medical Center Lihuili Hospital, Ningbo University, Zhejiang, China. Written informed consent was obtained from all subjects or their relatives.

### Magnetic resonance imaging data acquisition

Magnetic resonance imaging data were collected using a 3.0-T clinical MR image unit (Discovery MR750, GE Healthcare, Milwaukee, Wisconsin) using an eight-channel head coil. Conventional axial T2-weighted images had previously been obtained to rule out cerebral infarction or other lesions. Structural MRI scans were acquired using a sagittal three-dimensional (3D) T1-weighted sequence (repetition time, 7.4 ms; echo time, 3.2 ms; inversion time, 450 ms; flip angle, 12°; field of view, 25.6 × 25.6 mm; matrix, 256 × 256; slice thickness, 1 mm). A single-shot echo-planar imaging sequence was then used to acquire DTI images in the axial plane. Magnetic resonance images with 30 non-collinear diffusion gradients and without diffusion gradients were acquired (repetition time, 8175 ms; echo time, 80.8 ms; flip angle, 90°; field of view, 25.6 × 25.6 mm; matrix, 128 × 128; slice thickness, 2 mm; B factor, 1000 s/mm^2^).

### Data pre-processing and network construction

The topological properties of the brain were studied using the binary graph method G = (V, E), and nodes/vertices (V, E) were used to represent brain regions (i.e. ROI) and edges (E) between two nodes in the graph. To analyze complex networks, we applied a generalization of a simple graph called a weighted graph.

For graph construction, nodes and edges needed to be defined. The details of each node and edge were as follows:

#### Node definition

In this study, all image processing, including image registration, standardization, and custom templates to create space, was performed using PANDA software (PANDA, version 1.3.1, https://www.nitrc.org/projects/panda/)^37^. The T1-weighted image of each subject was co-registered with the B0 image in the DTI space. Then, the transformed T1 image was nonlinear transformed into the MNI152 T1 template in MNI space. The inverse transform was used to distort the Brainnetome Atlas (BNA) (http://atlas.brainnetome.org/) from the MNI space to the DTI native space. Finally, 105 cortical and 18 subcortical regions were obtained from each hemisphere. Then, ANTs (https://sourceforge.net/projects/advants/) software was used to co-register the 246 Gy matter (GM) regions masks into the individual diffusion space to complete the node definition of the brain structural network.

#### Edge definition

PANDA software, a matlab toolbox for analyzing brain diffusion images, was used to process DTI images. The steps included head motion and eddy current correction, removal of brain tissue, estimation of the probability distribution of dispersion direction using BedPostx, and probabilistic fiber tracking between two brain regions using Protrackx2, to obtain the probability value of the connection between brain regions, which represented the edge of the network. We averaged the mean probability values of the connected streamlines between two regions as the weights of the network edges. These steps resulted in a weighted WM network for each subject.

### Network analysis

We characterized the weighted global topological attributes (shortest path length L_p_, clustering coefficient C_p_, modularity, and small-worldness σ) and regional topological attributes (nodal efficiency E_nod_ and hubness score) of the WM networks using the brain graph package (https://cran.r-project.org/package=brainGraph) in R language (version: 3.6.3, https://www.r-project.org/). The traditionally used small-worldness is highly sensitive for classifying small-world networks. However, this measure is associated with low specificity, resulting in networks being classified as small-world when they are essentially random, with only minor clustering. Therefore, we performed a cluster-correction analysis of the small-worldness to better match the original description of small-world networks by Watts and Strogatz^38^.

### Connectivity analysis

A network-based statistic (NBS) approach was used to identify the specific altered WM connections associated with MA dependence. We first used a one-tailed test at each edge to determine the significant between-group differences in structural connection. A primary threshold (P < 0.05) was applied to define a set of suprathreshold edges. Then, we identified any connected subnetworks and their sizes (number of links). Under the null hypothesis of random group membership (5000 permutations), the empirical zero distribution of the maximum component size was used to obtain the statistical significance of each observed component size.

### Graph theory analysis and inter-group comparisons

To avoid errors caused by a single threshold and to facilitate comparisons of topological attributes between groups, the area under the curve (AUC) of each topological attribute within a threshold range was used to conduct a statistical analysis of network topology attributes^39^. The network consensus threshold range in this study was 0.001 to 0.01 with an interval of 0.001^40^.

The statistical significance level was set at P < 0.05. Each network topological attribute was compared to assess group differences using a general linear model (p < 0.05, permutation test, 10,000 times for global topological attributes and 5000 times for regional topological attributes).

The topological attributes with significant inter-group differences were examined using Pearson’s partial correlations with clinical parameters (duration of MA use, age at first MA use, HAMA score, BPRS score, and five-factor scores). Age, education, and cigarette smoking were used as covariate inputs to correct for their possible effects.

### SVM-based classification

The Least Absolute Shrinkage and Selection Operator (LASSO) is a regularization and variable selection algorithm implemented in the glmnet package in R (https://cloud.r-project.org/package=glmnet) for select an optimal feature subset from global and nodal topological properties, and perform 10 repeats of fivefold cross-validation. The selected features were used to build a linear SVM using the caret package in R (https://github.com/topepo/caret/). SVM is a supervised learning method, which has been widely used in statistical classification and regression analysis. It's mapping vectors to a higher dimensional space, where we set up a hyperplane with maximum spacing. Two hyperplanes are built parallel to each other on either side of the hyperplane separating the data, and the difference between the two types of data is maximized by calculating the hyperplane separating the two data optimally. This process is similar to that detailed in our previous work^31^. In short, the fivefold cross validation framework is applied to evaluate the performance of the classifier^41^. Before each cross-validation, scale the training dataset between 0 and 1 and use the obtained parameters to scale the test dataset^42,43^. Since the fivefold separation was random, we repeated the fivefold cross-validation 100 times. The presented performance was the average of 500 (fivefolds × 100) trials (expressed as mean values ± standard deviation). The only parameter C, which controls the trade-off between the whitespace width and the misclassification penalty, is set to the default value (C = 1).

Accuracy, sensitivity, specificity, and Kappa were calculated to quantify the cross-validation prediction performance of these classifiers. Specifically, accuracy was related to the proportion of subjects who were correctly classified as MA-dependent or HC (healthy control), and sensitivity and specificity were related to the proportion of individuals who were correctly classified as MA-dependent or HC. Kappa is similar to accuracy, except that it is normalized over a baseline of random probabilities on the dataset.

A one-tailed permutation test was used to assess the probability of obtaining a cross-validation precision value higher than that obtained by chance. All subjects were randomly relabeled and classified with fivefold cross-validation. The above process was repeated 5000 times, and the number of times the accuracy of the permuted label was higher than that of the true label was recorded. Then calculate the P perm value for the classification by dividing that number by 5000.

### Ethical approval

All procedures performed in studies involving human participants were in accordance with the ethical standards of the institutional and/or national research committee and with the 1964 Helsinki declaration and its later amendments or comparable ethical standards.

### Consent to participate

Written informed consent was obtained from all of the participants.

## Results

### Demographic and clinical characteristics

The demographic and clinical characteristics of the subjects are presented in Table 1. There were no statistically significant differences in age, education level, or the Fagerstrőm test for nicotine dependence (FTND) between patients with MA dependence and controls.

**Table 1:** Demographic and clinical characteristics of methamphetamine (MA)-dependent patients and normal controls (NCs).

Variables	Group		t	P
	MA	NC		
Sample size, n	46	46	–	–
Age (years) (mean ± SD)	34.9 ± 7.3	33.1 ± 10.9	0.727	0.471
Education (years)	13.20 ± 4.21	13.59 ± 3.67	− 0.331	0.740
FTND	6.30 ± 1.98	4.85 ± 2.35	0.497	0.505
Ages of the first MA use	30.50 ± 7.09	–	–	–
Total dose (g)	1065.91 ± 925.91		–	–
Brief Psychiatric Rating Scale	42.8 ± 11.04	–	–	–
Anxiety-depression factor	13 ± 3.57	–	–	–
Lack of vitality factor	8.43 ± 2.88	–	–	–
Thinking disorder factor	7.03 ± 2.33	–	–	–
Activity factor	7.3 ± 3.24	–	–	–
Hostility-suspicion factor	7 ± 2.99	–	–	–
Hamilton Anxiety Scale	22.39 ± 8.60	–	–	–

FTND, Fagerstrőm test for nicotine dependence.

### NBS analyses

There were no differences in WM connections between the MA-dependent group and the control group.

### Global topological attributes

**Figure 1:**
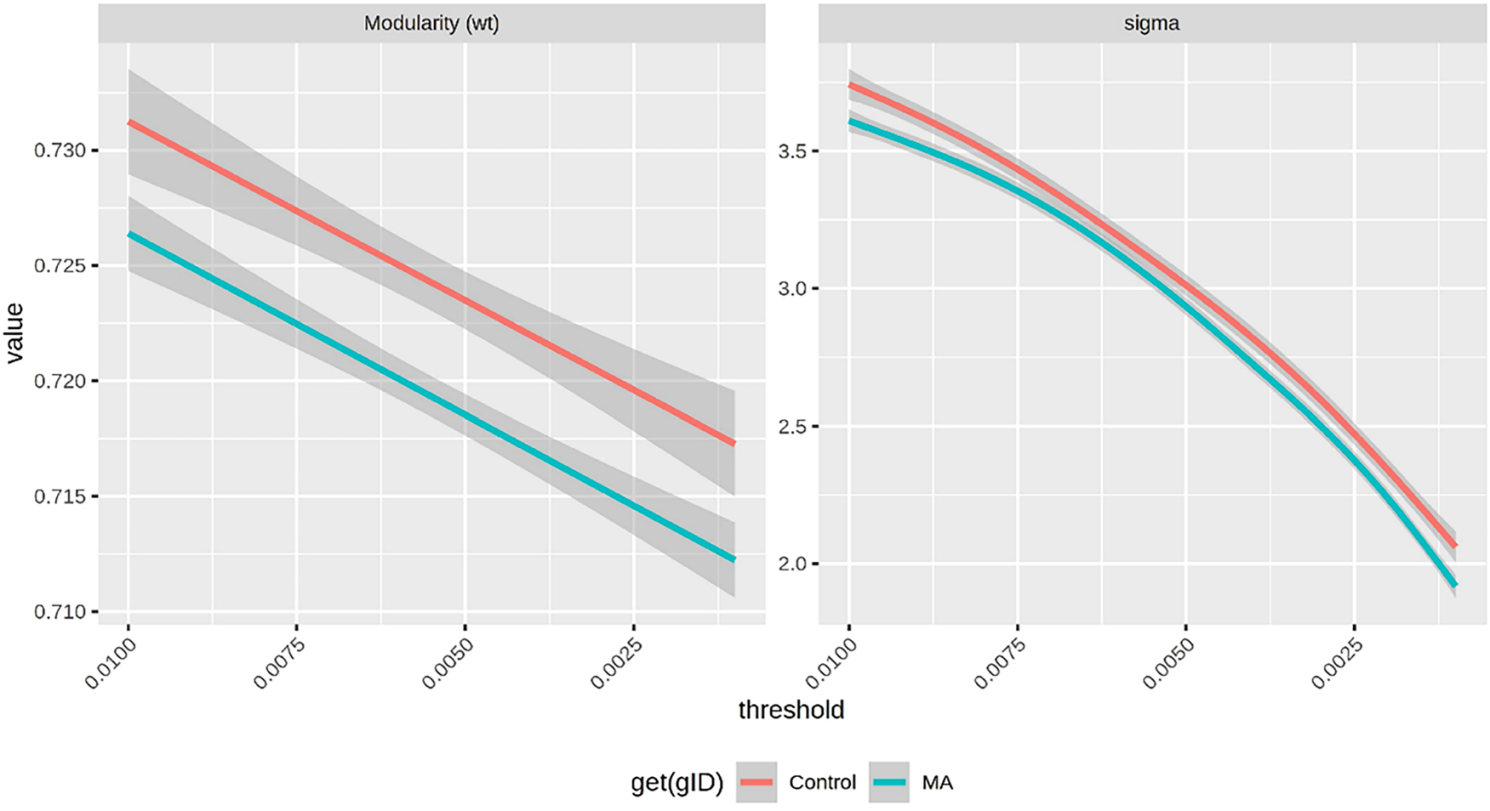
Network modularity and small-worldness (σ) in control and MA groups at each threshold. MA: methamphetamine-dependent patients; control: healthy controls.

Compared with the normal control group, the MA-dependent group exhibited significantly decreased small-worldness σ and reduced modularity of the WM network (Table 2) (Fig. S1 in Supplementary Materials). In addition, as shown in Fig. 1, at each threshold, the small-worldness σ and modular values of the MA-dependent group were lower than those of the control, and the σ values of both groups were greater than 1, which indicated that the MA-dependent group and the control group showed small-worldness in the WM networks.

**Table 2:** Global topological attributes of brain WM structural networks in the methamphetamine (MA)-dependent patients (MA) and the normal controls (Control).

Network topological attributes	Contrast	P value	Cohen’s d
σ	Control > MA	0.008	0.5107
C_p_	Control > MA	0.734	− 0.1309
L_p_	Control > MA	0.060	0.3153
Q_m_	Control > MA	0.023	0.4213

σ = small-wordness; C_p_ = weighted clustering-coefficient; L_p_ = weighted characteristic path length; Q_m_ = weighted modularity.

Cohen’s d indicates the value of effect size. The small, medium and large levels of the effect size are 0.2, 0.5 and 0.8, respectively, according to Cohen’s definition.

### Regional topological attributes

**Figure 2:**
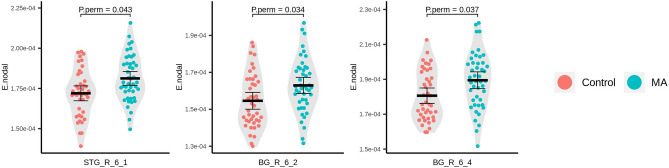
Brain regions with significant differences in nodal efficiency between methamphetamine-dependent patients and healthy controls. STG_R_6_1 = the weighted nodal efficiency of the right medial superior temporal gyrus; BG_R_6_2 = the weighted nodal efficiency of the globus pallidus; BG_R_6_4 = the weighted nodal efficiency of the ventromedial putamen. MA: methamphetamine-dependent patients; control: healthy controls.

In the MA-dependent group, E_nod_ was significantly increased in the right medial superior temporal gyrus (mSTG), the right pallidum, and the right ventromedial putamen (Fig. 2).

**Figure 3:**
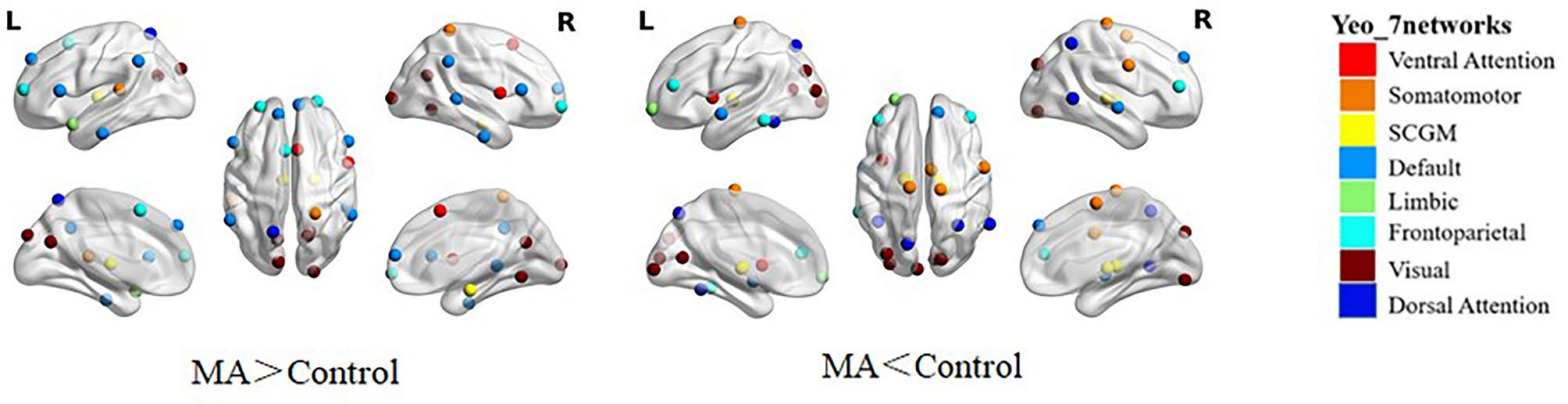
Brain regions with significant differences in hubness scores between methamphetamine-dependent patients and healthy controls. Different colors of brain nodes represent different subnetworks of the Yeo_7networks. The statistical criterion for between-group differences was set at p < 0.005 after 5,000 permutation test. Detailed brain regions are described in Table S1, Supplementary Materials. MA: methamphetamine-dependent patients; control: healthy controls. SCGM = Subcortical gray matter.

As shown in Fig. 3 (Table S1 in Supplementary Materials), the control group had the higher hubness scores in 26 regions compared with the MA-dependent group, most of which belonged to the dorsal attention network (DAN) and the somatomotor system. The MA-dependent group had the higher hubness scores in 25 regions compared with the control group, these regions were mainly located in the default-mode network (DMN).

### Clinical/demographic correlations

At the global level, small-worldness σ was negatively correlated with BPRS total scores and hostility/suspicion factor scores in patients with MA dependence.

**Figure 4:**
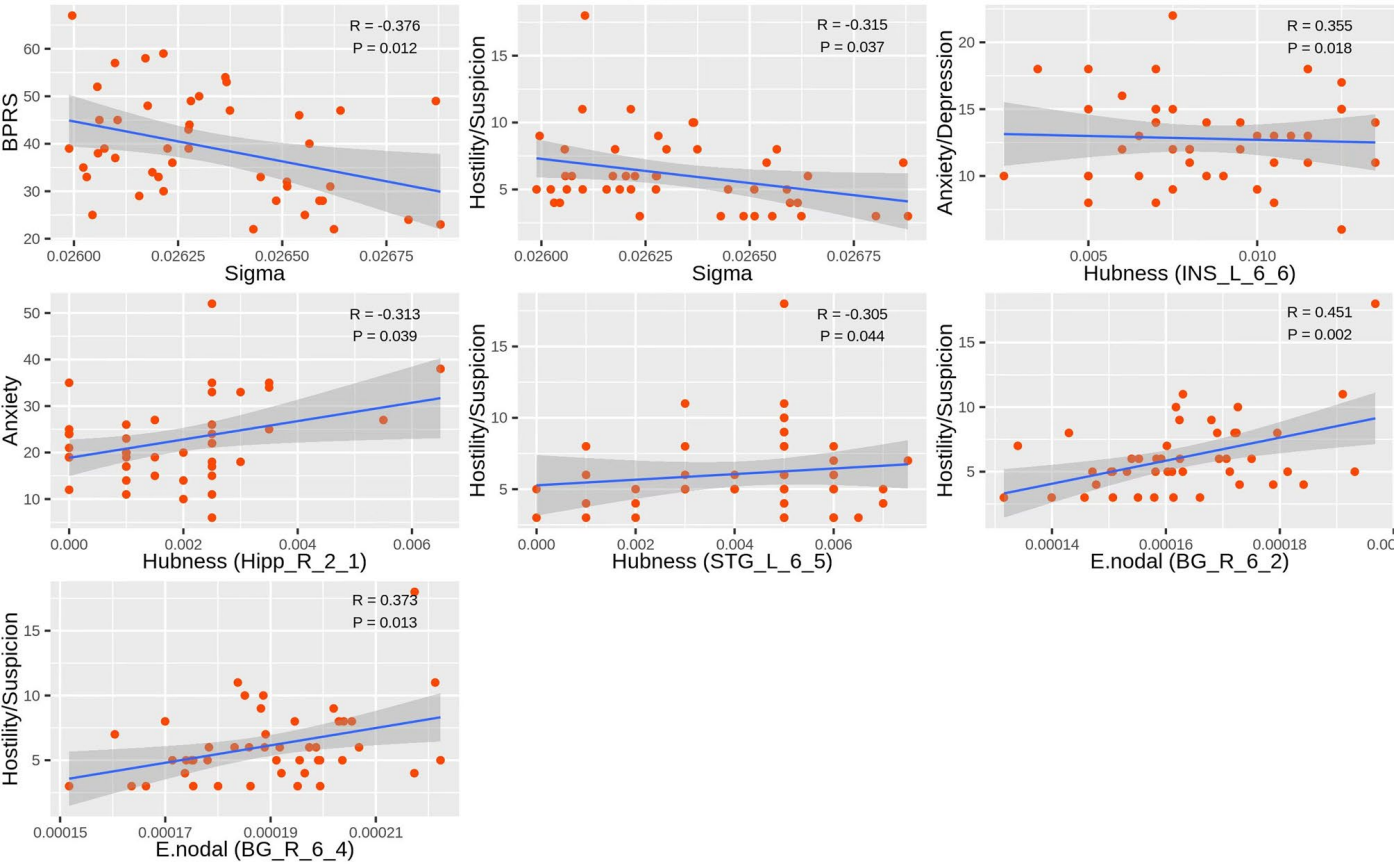
The correlation between weighted topological attributes and HAMA scores or Brief Psychiatric Rating Scale (BPRS) (total scores or factor scores) in methamphetamine-dependent patients. Sigma = small-worldness scalar; INS_L_6_6 = the hubness scores of the left dorsal dysgranular insula; Hipp_R_2_1 = the hubness scores of the rostral hippocampus; STG_L_6_5 = the hubness scores of the left superior temporal gyrus caudal area; BG_R_6_2 = the weighted nodal efficiency of the globus pallidus; BG_R_6_4 = the weighted nodal efficiency of the ventromedial putamen.

At the regional level, the hubness scores of the left superior temporal gyrus, and the nodal efficiency of the right pallidum and right putamen, were positively correlated with the severity of positive psychotic symptoms in patients with MA dependence. The severity of anxiety/depression was negatively correlated with the hubness scores of the left insula, and the severity of anxiety was positively correlated with the hubness score of the right hippocampus (Fig. 4).

### SVM classification

Based on the general linear model, 57 network topology attributes with evident inter-group differences are obtained. After performing LASSO filtering features, 12 optimal topology attributes are selected as features to construct a machine training model using a linear SVM. The SVM exhibited excellent performance, with cross-validated prediction accuracy, sensitivity, specificity, and kappa values of 98.09% ± 2.59%, 98.24% ± 4.00%, 97.94% ± 4.26%, and 96.18% ± 5.19%, respectively. (P.perm < 0.001) (for detailed feature importance of 12 topological attributes values, see Table 3).

**Table 3:** The importance of 12 topological attributes values in the classifying process.

Topological attributes	Weights
Hubness (left superior parietal lobule caudal area)	0.215
Hubness (right precentral gyrus head and face region)	0.273
Hubness (right caudal lingual gyrus)	0.329
Hubness (right medial superior occipital gyrus)	0.0498
Hubness (right medial superior frontal gyrus)	0.136
Hubness (left inferior parietal lobule caudal area)	0.312
Hubness (left dorsal dysgranular insula)	0.14
Hubness (right occipital polar cortex)	0.2466
Hubness (right precentral gyrus tongue and larynx region)	0.258
Hubness (right inferior frontal gyrus caudal area)	0.2137
Hubness (right inferior parietal lobule caudal area)	0.044
Hubness (left dorsomedial parietooccipital sulcus)	0.4139

Hub (i) = weighted hub of node I; Weights = the importance of topological attributes.

## Discussion

The present study showed altered topological organization of the WM network in MA-dependent patients. The main findings for patients with MA dependence were as follows: (1) there were no differences in WM connections between the MA-dependent group and the control group.; (2) significantly decreased small-worldness and modularity in MA-dependent patients; (3) the regions with increased E_nod_ were located in the right mSTG, right pallidum, and right ventromedial putamen in MA-dependent patients; (4) the MA-dependent group had higher hubness scores in the DMN compared with the control; (5) small-worldness σ was negatively correlated with BPRS total scores and hostility/suspicion factor scores in patients with MA dependence. The hubness scores of the left superior temporal gyrus, and the nodal efficiency of the right pallidum and right putamen, were positively correlated with the severity of positive psychotic symptoms in patients with MA dependence. The severity of anxiety/depression was negatively correlated with the hubness scores of the left insula, and the severity of anxiety was positively correlated with the hubness scores of the right hippocampus; (6) a classifier trained on network topological attributes had excellent classification performance for MA dependence and suggested that these topological attributes could be promising features for diagnosis of MA dependence. These findings improved our understanding of the neuropathological mechanisms of development of MA dependence at the level of a large-scale whole-brain WM networks.

### Abnormal global topological organization in WM network

The small-world network topology model is characterized by a high clustering coefficient and a small shortest path length^44^. Small-worldness reflects the optimal balance between local specialization and global integration of brain regions and the ability of the brain to adapt to various external stimuli^45^. Consistent with the results of previous studies of individuals with MA dependence^46^, the whole-brain structural network of the MA-dependent group and that of the controls had conserved small-worldness, but the brain network of the patients with MA dependence was not optimally configured. This suggested that the function of the small-world network in patients with MA dependence patients was impaired and tended toward randomness. In addition, lower small-world score was associated with higher BPRS total scores and higher hostility/suspicion disorder factor scores in our study. Several studies have reported that abuse of MA can lead to greater hostility than controls^47,48^. Therefore, the transformation of the structural network to a random network in patients with MA dependence may be the initiating factor in subsequent development of positive psychotic symptoms such as hostility/suspicion.

At the global level, we observed lower modularity in patients with MA dependence that in controls. Modularity measures the division of a network into separate modules^44^. A module is defined as having denser connections between nodes within the module but sparser connections with nodes outside the module. Lower modularity in patients with MA dependence may indicate fewer connections within the module, but more connections between modules. Yafei et al.^49^ found that the degree of modularity in individuals with MA addiction was significantly lower than that of healthy controls in a resting-state MRI study. This was consistent with our results. Modular network organization is adaptable and can evolve^50^. Chronic MA abuse leads to reorganization of brain networks. For example, the exchange of information between modules (systems) was abnormally increased in patients with MA. During long-term exposure to drugs or drug-related cues, the sensory system (visual or auditory) rapidly transmits relevant information to the memory system, leading to overactivation of the reward and motivational systems, which results in compulsive drug use. Similar reductions in modularity have also been observed in patients with cognitive deficits^51,52^. These results indicated that impaired cognitive function in individuals with MA dependence may be related to disruption of cognition-related intramodular connectivity.

### Between-group differences in node efficiency

We observed significantly increased E_nod_ in the right mSTG, the right pallidum, and the right ventromedial putamen in individuals with MA dependence compared with that in normal controls. The superior temporal cortex (gyrus and sulcus) is part of a complex facial processing system related to emotional perception^53^ and regulation of response to negative visual social stimuli^54^. Abnormal superior temporal gyrus function is often accompanied by psychological changes^55^ such as anxiety, auditory hallucinations, and delusions. The findings of Tsujii et al.^56^ showed that abnormal superior temporal gyrus function was related to impaired emotional control and behavioral inhibition. They suggested that changes in the superior temporal gyrus may result in cognitive symptoms and social disorders in some patients with bipolar disorder. Patients with MA dependence often develop mood regulation disorders^57^ and experience abnormal social cognitive function^58^. Previous studies have reported superior temporal gyrus abnormalities in patients with MA, such as abnormal volume^59^ and changes in regional homogeneity^60^. Increased E_nod_ of the superior temporal gyrus in our study may further indicate the importance of this region in emotional processing network disorder. The pallidum is a point of convergence for limbic reward signals and the intermediate stages of various cognitive, emotional, and motor processes. It is a central site for coding and promoting reward learning, enjoyment, and motivation^61^. In patients with MA dependence, increased E_nod_ in the pallidum results in enhancement of reward and motivation through periodic bursts of excitation and hedonic stimuli, leading to compulsive drug intake. The putamen is a part of the mesocorticolimbic reward circuit. Previous studies found that MA- and cocaine-induced cravings were associated with activation of the putamen^62,63^. Drug craving is the main motivation for increased drug use, and is thought to be a significant factor in relapse. Cravings are persistent in patients with MA dependence, which suggests that the putamen is a critical component of addiction-related networks.

### Identification of network hubness

Hubness were defined as nodes with high nodal centrality, which is an indicator of the importance of nodes in networks of interacting brain regions. In our study, the MA-dependent group had higher hubness scores in 25 regions compared with the control group, these regions were mainly located in the default-mode network (DMN), most of which were located in the DMN. The locations of these DMN regions included the superior frontal gyrus, the inferior frontal gyrus, and inferior parietal lobule. The DMN is a cluster of brain regions that are spontaneously active in resting states and associated with internal directed cognition. Many neuropsychiatric disorders are associated with DMN dysfunction^64,65^. Changes in resting state functional connectivity in the DMN have been observed in individuals with MA dependence^66^. The frontal brain regions of the DMN are involved in excitatory and inhibitory regulation of cravings associated with addiction^67,68^. The inferior parietal lobule was shown to simulate future behavior using mnemonic imagery-based processes^69^. Therefore, reorganization of hubness distribution in the DMN may reflect enhanced memory for drug-related cues, strong drug craving, and drug-seeking behavior in individuals with MA dependence.

In contrast, the patients with MA dependence in our study had lower hubness scores in the DAN, the somatomotor system. Recent studies of substance addiction have shown varying degrees of attention disorders in individuals with addiction, as evidenced by attention bias for substance-related cues^70,71^. Attention is based on choosing between conflicting needs at different processing levels and in different cognitive fields using limited resources. The DAN plays an important role in top-down (proactive) attention processing^72^. Patients with MA dependence patients lose DAN hubness for multiple reasons. Long-term use of MA may damage the DAN, the lack of attention and indifference to surrounding things are often accompanied by external symptoms (e.g. apathy, disorientation, etc.) of MA-dependent patients. Impairment of the DAN may promote decreased spatial attention ability. Methamphetamine is a potent psychoactive stimulant that can increase physical strength and energy in small doses. However, long-term use of large doses can result in inhibition of cholinergic receptors in skeletal muscle motor endplates, resulting in the decline of body vitality and the weakening of the body's response to external stimuli. Therefore, the somatomotor system showed decreased activity in patients with MA dependence compared with controls.

### Correlation between topological attributes and clinical variables

We used correlation analysis to gain a preliminary understanding of factors associated with onset and progression of psychotic symptoms observed in individuals with MA dependence. Our results showed that changes in nodal efficiency in the left superior temporal gyrus, and in hubs within the right pallidum and right putamen, were positively correlated with positive psychotic symptoms. The temporal lobe is the perceptual center in humans, and positive symptoms of schizophrenia are strongly associated with sensory perceptions, such as hallucinations^73,74^. Sabri et al.^73^ analyzed brain imaging data from 24 patients with schizophrenia who were not treated with antipsychotic drugs, and found that delusions, hallucinatory behaviors, suspicion, and victimization were positively correlated with rCBF (regional cerebral blood flow) in the left temporal lobe. This study agreed with our finding of a significant positive association between the hubness scores of the left superior temporal gyrus and BPRS factor scores, especially positive symptoms. Galati et al.^75^ reported a case of paranoid schizophrenia with carbon monoxide poisoning-induced pallidum damage, which resulted in complete remission of some positive symptoms, such as paranoia. This indicated that the pallidum may be associated with positive symptoms of schizophrenia. In patients with schizophrenia, the volume^76^, nodal efficiency^77^, betweenness centrality^39^, and the amplitude of low-frequency fluctuations^78^ in the putamen were abnormal. Hong et al.^76^ found that putamen volume was associated with positive symptoms of psychosis, and speculated that putamen volume might be an indicator of risk and clinical course prediction for development of clinical psychosis. These studies support our findings that these psychiatric symptoms may not be caused by abnormalities in single brain regions or neural pathways, but by a combination of multiple neural pathways or brain regions. Future studies of MA-induced psychiatric symptoms should focus on networks of neural pathways and brain regions.

Anxiety is one of the most common psychiatric symptoms in MA users^79^. Individuals with anxiety are more prone to anxiety symptoms in response to uncertainty, and processing of uncertain information is an important, but often neglected factor in anxiety. The insula receives interoceptive information. Paulus and Stein proposed that “the difference between the insula in determining the internal perception expected from the stimulus and the prediction of its results is very important”^80^. The difference between the observed and expected body state may lead to an anxiety state, and the emotional, cognitive, and behavioral components representing anxiety may be a result of a change in this prediction signal^80^. The hippocampus is the brain structure that processes emotions, integrates sensory, emotional, and cognitive components of pain, and processes information about the body. The hippocampus exerts tonic inhibitory control over the hypothalamic stress-response system. Considering the association between the hubs in the above-mentioned area (the insula and hippocampus) and the anxiety scores in our study, we hypothesize that the long-term effects of MA lead to adaptive changes in the central role of these regions in patients with MA dependence. When individuals see drug-related cues (such as videos or pictures), relevant information is transmitted to the brain, which results in generation of internal feelings (such as ' hunger and thirst '). The insula and the hippocampus process and integrate internal feelings, and the hippocampus inhibits the stress system of the hypothalamus, and prevents release of γ-aminobutyric acid (GABA), resulting in anxiety symptoms^81^.

### SVM classifier

In the present study, we demonstrated that the SVM approach combined with graph-derived measures showed an excellent ability to distinguish patients with MA dependence from control subjects based on their WM network topological features.

In addition, we extracted the weight value of each feature in the classifier. Among these graph theory-related features, the hubness of the right superior frontal gyrus, the left superior parietal lobule, the left inferior parietal lobule, the right occipital pole cortex, the right precentral gyrus, left parietooccipital sulcus, and right lingual gyrus had larger weight values. The superior frontal gyrus is located in the upper part of the prefrontal cortex, and is an important region involved in tasks such as movement, working memory, and cognitive control^82^. The precentral gyrus belongs to the primary motor cortex and mainly controls somatic motor behavior. Kim et al.^83^ used voxel-based morphometry (VBM) to study the gray matter integrity of both long-term (30.6 months) and short-term (2.6 months) abstinent subjects with MA dependence. They found that density of the prefrontal cortex was lower in the subjects who abstained from MA compared with the control group. In addition, Kim et al.^84^ found that MA use resulted in persistent low metabolism in frontal WM and impaired executive function of the frontal lobe. The parietal lobe is primarily involved in cognition, attention, and decision-making^85^. Yang et al.^60^ observed increased parietal cortex thickness in abstinent users of MA. Impaired cognitive control in individuals with MA dependence can be understood as a result of disordered regional networks in the prefrontal and parietal cortices. The occipital lobe is the key visual center, and is responsible for processing and synthesis of visual information. The parieto-occipital sulcus is located in the anterior border of the occipital lobe and is an important part of the brain functional area, which belongs to the visual motor processing area. The lingual gyrus is located in the primary visual cortex and plays an important role in visual perception and visual memory processing. In individuals with MA dependence, long-term search for drugs or drug-related clues leads to the activation of vision-related functional areas. These findings indicated that diagnosis of MA may be closely related to cognitive control functions and the visual system, and that these features may be potential predictive markers of MA abuse.

### Limitation and future directions

Our study was subject to several limitations. First, the small sample size did not allow for comparisons of differences in brain structural networks between subgroups (for example, between MA dependence with or without psychotic symptoms) or evaluation of correlation with the BPRS scale, which may affect the generalizability of the study results. A larger sample size is needed for future subgroup studies. Furthermore, the small sample size may have resulted in overfitting during cross-validation for machine learning-based classification. To test the generalizability of the model, future studies should train the classifier on a larger dataset and validate the classifier on an independent dataset that was not used in any training iteration. Second, as DTI depends on the diffusion parameters of water and its spatial resolution is relatively low compared to the actual size of nerve fibers, DTI has difficulty identifying complex fibrous tissues, such as crossing, converging, and diverging fibers^86,87^. However, DTI is currently one of few tools available for in vivo evaluation of human brain structural networks. To increase the sensitivity of fiber reconstruction, we used a probabilistic tractography method to reconstruct the networks. Use of probabilistic tractography with a crossing fiber model improved the sensitivity for capturing the complexity of neural fiber organization^88^. Third, as this was a cross-sectional study, we cannot determine whether the differences in topological attributes were a consequence of MA exposure or were present as predisposing factors for development of addiction. Genetic and longitudinal imaging studies are needed to resolve this issue. Last but not the least, since there is no widely accepted standard to construct the cortex and subcortical area of the brain, the nodes of the structural network are defined by predefined templates, such as automatic anatomical marker map. It is well known that the topological properties of brain networks constructed with different brain maps are not consistent^89,90^. In future research, it will be important to study the brain network with more advanced segmentation methods—for example, dividing the brain into smaller and more compact areas^91^, or trying to define functional areas based on resting state or task induced response^92^.

## Conclusion

In summary, the present study provided evidence that MA dependence was associated with abnormal WM network topological attributes, such as disturbed small-worldness and altered nodal efficiency, which could be used as features for construction of a machine learning-based classifier to accurately diagnose MA dependence.

## Supplementary Information

Supplementary Information.

## Data Availability

The datasets analyzed during the current study are not publicly available but are available from the corresponding author on reasonable request.
