# Supplementary material for: Aberrant topology of white matter networks in patients with methamphetamine dependence and its application in support vector machine-based classification
Source: Sci Rep. 2023 Apr 28;13:6958. doi: 10.1038/s41598-023-33199-8 (PMC10147725; doi:10.1038/s41598-023-33199-8)
Supplement: Supplementary file 1 — Supplementary Information. [file 41598_2023_33199_MOESM1_ESM.docx]

**Aberrant topology of white matter networks in patients with methamphetamine dependence and use of support vector machine‐based classification**

**Ping Cheng^1^, Yadi Li^1^, Gaoyan Wang^1^, Haibo Dong^1^, Huifeng Liu^2^, Wenwen Shen^2^, Wenhua Zhou^2^**

^1^Department of Radiology, Ningbo Medical Treatment Center Lihuili Hospital, Ningbo University, Ningbo, Zhejiang, China

^2^Department of Psychiatry, Ningbo Kangning hospital, Ningbo University, Ningbo, Zhejiang, China

*** Correspondence:**

1. Yadi Li, Ningbo Medical Treatment Center Lihuili Hospital, 57# Xing Ning road, Ningbo, Zhejiang, China. Email: liyadi2010@126.com; 2. Wenhua Zhou, Ningbo Kangning hospital, 1# Zhuangyu South Road, Ningbo, Zhejiang, China. Email: whzhou@vip.163.com

**Keywords:** Methamphetamine, Substance addiction, Diffusion tensor imaging, Graph theory analysis, Machine learning

Fig.S1 Brain regions with significant differences in global topological attributes between methamphetamine-dependent patients and healthy controls.


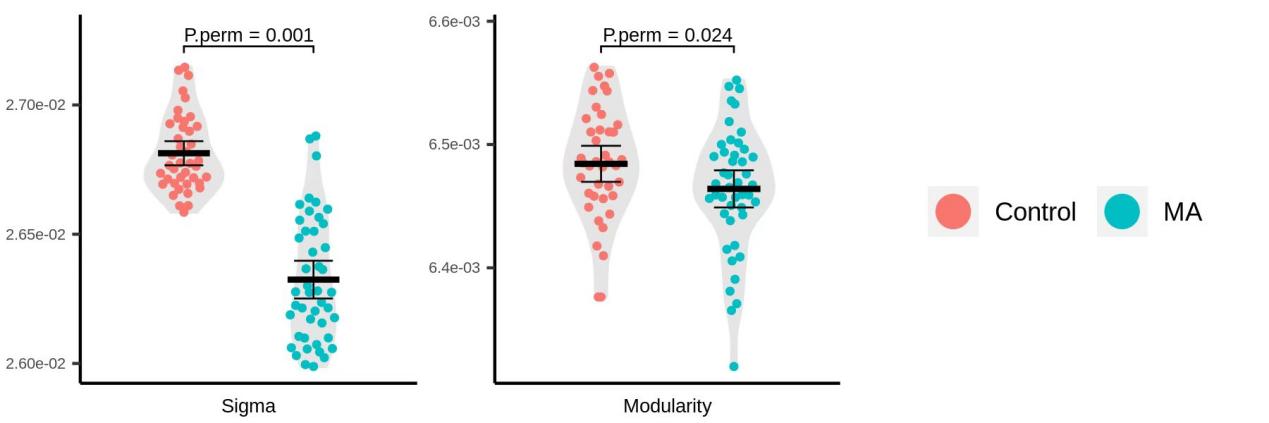


MA: methamphetamine-dependent patients; control: healthy controls.

Table S1

Hubness regions in patients with methamphetamine dependence (MA) and the normal controls (Control).

|  | **Hubness region** | **Subnetwork** | **t-value** | **P****-value** |
| --- | --- | --- | --- | --- |
| **Control＞MA** | Right superior frontal gyrus (medial area 6） | Somatomotor | 4.4868 | 0.0022 |
|  | Right superior frontal gyrus (lateral area 9) | DMN | 11.92968 | 0.0002 |
|  | Right middle frontal gyrus (ventral area) | Frontoparietal | 4.0737 | 0.0086 |
|  | Left middle frontal gyrus (ventral area) | Frontoparietal | 5.3830 | 0.0004 |
|  | Left middle frontal gyrus (lateral area10) | Limbic | 5.8277 | 5.8277 |
|  | Right precentral gyrus (area 4 (head and face region)) | Somatomotor | 12.4797 | 0.0002 |
|  | Right precentral gyrus (area 4 (trunk region)) | Somatomotor | 4.6394 | 0.0012 |
|  | Left precentral gyrus (area 4 (trunk region)) | Somatomotor | 5.0315 | 0.0006 |
|  | Right superior temporal gyrus (rostral area 22) | DMN | 4.2240 | 0.0054 |
|  | Left superior temporal gyrus (rostral area 22) | DMN | 3.5682 | 0.0390 |
|  | Right middle temporal gyrus (dorsolateral area 37) | DAN | 5.1301 | 0.0006 |
|  | Left inferior temporal gyrus (caudolateral of area 20) | Frontoparietal | 9.6946 | 0.0002 |
|  | Left fusiform gyrus (lateroventral area 37) | DAN | 5.4068 | 0.0004 |
|  | Left superior parietal lobule (caudal area 7) | DAN | 12.1548 | 0.0002 |
|  | Right superior parietal lobule (intraparietal area 7) | DAN | 5.0642 | 0.0006 |
|  | Left inferior parietal lobule (caudal area 39) | Visual | 4.0656 | 0.0086 |
|  | Left insular gyrus (dorsal dysgranular insula) | VAN | 12.1843 | 0.0002 |
|  | Right medioventral occipital cortex (caudal lingual gyrus) | Visual | 9.9553 | 0.0002 |
|  | Left medioventral occipital cortex (caudal cuneus gyrus) | Visual | 6.4464 | 0.0002 |
|  | Left medioventral occipital cortex (ventromedial parietooccipital sulcus) | Visual | 5.5856 | 0.0002 |
|  | Left lateral occipital cortex (middle occipital gyrus) | Visual | 4.9471 | 0.0006 |
|  | Right lateral occipital cortex (medial superior occipital gyrus) | Visual | 9.3701 | 0.0002 |
|  | Right thalamus (pre-motor thalamus) | SCGM | 6.7165 | 0.0002 |
|  | Left thalamus (pre-motor thalamus) | SCGM | 4.7853 | 0.0008 |
|  | Right thalamus (sensory thalamus) | SCGM | 3.8355 | 0.0182 |
|  | Left thalamus (lateral pre-frontal thalamus) | SCGM | 4.0421 | 0.0098 |
| **MA＞Control** | Right superior frontal gyrus (medial area 8) | VAN | 5.0035 | 0.0004 |
|  | Left superior frontal gyrus (medial area 8) | Frontoparietal | 7.6885 | 0.0002 |
|  | Left superior frontal gyrus (lateral area 9) | DMN | 7.2392 | 0.0002 |
|  | Right superior frontal gyrus (medial area 10) | DMN | 4.5770 | 0.0010 |
|  | Left middle frontal gyrus (area 46) | Frontoparietal | 4.5962 | 0.0010 |
|  | Right middle frontal gyrus (lateral area 10) | Frontoparietal | 5.6835 | 0.0002 |
|  | Right inferior frontal gyrus (caudal area 45) | DMN | 13.5532 | 0.0002 |
|  | Left inferior frontal gyrus (caudal area 45) | DMN | 4.0424 | 0.0080 |
|  | Right precentral gyrus (area 4 (tongue and larynx region)) | VAN | 9.8611 | 0.0002 |
|  | Left superior temporal gyrus (area 41/42) | Somatomotor | 6.3365 | 0.0002 |
|  | Left superior temporal gyrus (lateral area 38) | Limbic | 6.3509 | 0.002 |
|  | Right inferior temporal gyrus (intermediate lateral area 20) | DMN | 4.3001 | 0.0030 |
|  | Left inferior temporal gyrus (intermediate lateral area 20) | DMN | 5.4760 | 0.0002 |
|  | Right posterior superior temporal sulcus (rostroposterior superior temporal sulcus) | DMN | 8.1400 | 0.0002 |
|  | Left superior parietal lobule (rostral area 7) | DAN | 6.1042 | 0.0002 |
|  | Right superior parietal lobule (postcentral area 7) | Somatomotor | 4.3728 | 0.0022 |
|  | Right inferior parietal lobule (caudal area 40) | DMN | 14.5468 | 0.0002 |
|  | Left inferior parietal lobule (caudal area 40) | DMN | 9.7291 | 0.0002 |
|  | Right precuneus (dorsomedial parietooccipital sulcus) | Visual | 9.7337 | 0.0002 |
|  | Left precuneus (dorsomedial parietooccipital sulcus) | Visual | 6.0438 | 0.0002 |
|  | Right medioventral occipital cortex (rostral lingual gyrus) | Visual | 4.4561 | 0.0012 |
|  | Right lateral occipital cortex (occipital polar cortex) | Visual | 12.4666 | 0.0002 |
|  | Left lateral occipital cortex (medial superior occipital gyrus) | Visual | 7.6243 | 0.0002 |
|  | Right rostral hippocampus | SCGM | 5.7693 | 0.0002 |
|  | Left thalamus (medial pre-frontal thalamus) | SCGM | 6.8036 | 0.0002 |

DMN= the default mode network; DAN= the dorsal attention network; VAN=Ventral Attention network; SCGM=Subcortical gray matter

Table S2

The definitions and interpretations of global/nodal topological attributes in the current study.

| **Network topological attributes** | | **Definitions** | **Descriptions** |
| --- | --- | --- | --- |
| Global topological attributes | Small-worldness | $\boldsymbol{\sigma}=\frac{\boldsymbol{\gamma}}{\boldsymbol{\lambda}}=\frac{{\boldsymbol{C}_{\boldsymbol{p}}^{\boldsymbol{real}}}/{\boldsymbol{C}_{\boldsymbol{p}}^{\boldsymbol{rand}}}}{{\boldsymbol{L}_{\boldsymbol{p}}^{\boldsymbol{real}}}/{\boldsymbol{L}_{\boldsymbol{p}}^{\boldsymbol{rand}}}}$ | is the mean clustering coefficient of 100 matched random networks., is the mean clustering coefficient of 100 matched random networks. 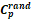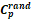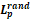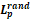 Small-world organization reflects an optimal balance of functional integration and segregation. A network is said to be small-world if γ>>1 and λ≈1 or σ>> 1 |
|  | Clustering coefficient | $C_{P}=\frac{1}{N}\sum_{j\neq i} \frac{E_{i}}{D_{nod}\left( i \right)(D_{nod}\left( i \right)-1)/2}$ | D_nod_ (i) is the degree of node i, E_i_ is the number of edges in the subgrap$在此处键入公式。$h of node i and N is the number of nodes in the network. Cp reflects the prevalence of clustered connectivity around individual nodes. |
|  | Characteristic path length | $p_{=}\frac{1}{1/{(N(N-1))\sum_{i\neq j} 1/{L_{ij}}}}$ | L_ij_ is the shortest path length between nodes i and j. Paths are sequences of distinct nodes and links in the network to represent potential routes of information flow between pairs of brain regions. The lengths of paths estimate the potential for integration between brain regions, with shorter paths  implying stronger potential for integration |
| Regional topological attributes | Nodal efficiency | $E_{\mathrm{nod}}(i)=\frac{1}{N-1}\sum_{j\neq i} \frac{1}{L_{\mathrm{ij}}}$ | The nodal efficiency is used for describing the nodal (regional)  characteristics of the WM structural network. |
|  | Betweenness centrality | $\mathbf{Q}\mathbf{=}\frac{\mathbf{1}}{\mathbf{2m}}\sum_{\mathbf{ij}} \left[ \mathbf{A}_{\mathbf{ij}}\mathbf{-}\frac{\mathbf{k}_{\mathbf{i}}\mathbf{k}_{\mathbf{j}}}{\mathbf{2m}} \right]\boldsymbol{\delta}\mathbf{(}\mathbf{C}_{\mathbf{i}}\mathbf{C}_{\mathbf{j}}\mathbf{)}$ | L_hj_(i) represents the number of shortest paths from h to j through node i. L_hj_ is the shortest path length between nodes h and j. Betweenness centrality represents the importance of a particular node for network communication and is  conceptualized by the number of shortest paths between any two  nodes in the network that has to pass through that particular node. |
